# Large-scale proteomic identification of S100 proteins in breast cancer tissues

**DOI:** 10.1186/1471-2407-10-476

**Published:** 2010-09-03

**Authors:** Patrizia Cancemi, Gianluca Di Cara, Nadia Ninfa Albanese, Francesca Costantini, Maria Rita Marabeti, Rosa Musso, Carmelo Lupo, Elena Roz, Ida Pucci-Minafra

**Affiliations:** 1 Dipartimento di Oncologia Sperimentale e Applicazioni Cliniche (DOSAC) Università di Palermo, Palermo, Italy; 2 Ospedale La Maddalena D.O. III livello, Palermo, Italy; 3 Centro di Oncobiologia Sperimentale (COBS), Palermo, Italy

## Abstract

**Background:**

Attempts to reduce morbidity and mortality in breast cancer is based on efforts to identify novel biomarkers to support prognosis and therapeutic choices. The present study has focussed on S100 proteins as a potentially promising group of markers in cancer development and progression. One reason of interest in this family of proteins is because the majority of the S100 genes are clustered on a region of human chromosome 1q21 that is prone to genomic rearrangements. Moreover, there is increasing evidence that S100 proteins are often up-regulated in many cancers, including breast, and this is frequently associated with tumour progression.

**Methods:**

Samples of breast cancer tissues were obtained during surgical intervention, according to the bioethical recommendations, and cryo-preserved until used. Tissue extracts were submitted to proteomic preparations for 2D-IPG. Protein identification was performed by N-terminal sequencing and/or peptide mass finger printing.

**Results:**

The majority of the detected S100 proteins were absent, or present at very low levels, in the non-tumoral tissues adjacent to the primary tumor. This finding strengthens the role of S100 proteins as putative biomarkers. The proteomic screening of 100 cryo-preserved breast cancer tissues showed that some proteins were ubiquitously expressed in almost all patients while others appeared more sporadic. Most, if not all, of the detected S100 members appeared reciprocally correlated. Finally, from the perspective of biomarkers establishment, a promising finding was the observation that patients which developed distant metastases after a three year follow-up showed a general tendency of higher S100 protein expression, compared to the disease-free group.

**Conclusions:**

This article reports for the first time the comparative proteomic screening of several S100 protein members among a large group of breast cancer patients. The results obtained strongly support the hypothesis that a significant deregulation of multiple S100 protein members is associated with breast cancer progression, and suggest that these proteins might act as potential prognostic factors for patient stratification. We propose that this may offer a significant contribution to the knowledge and clinical applications of the S100 protein family to breast cancer.

## Background

Breast cancer is still one of the most frequent forms of cancer in women. Unfortunately, the biological and clinical evolution of this type of cancer is not easily predictable since there are several types that behave differently among patients. This biological heterogeneity is consistent with observed varied responses to therapies across patient populations. For this reason the search for new biological markers to support prognosis and therapeutic options remains an open field in oncology research.

One class of proteins that is emerging as a potentially important group of markers in cancer development and progression is the S100 family. S100 are small, acidic-Ca^2+ ^binding proteins, found exclusively in vertebrates. The first member was identified in the nervous system by Moore in 1965 [1]. The S100 name is based on the observation that they are soluble in 100% saturated ammonium sulfate at neutral pH; at least 25 members of the S100 protein family are recognized in human. Twenty one of them (S100A1-S100A18, trichohylin, fillagrin, repetin) are coded by genes clustered at chromosome locus 1q21 (known as the epidermal differentiation complex), while the other genes belonging to the subfamilies of S100B, S100P, S100Z and S100G, are respectively located at chromosome loci 21q22, 4p16, 5q14 and Xp22 [2]. S100 proteins form homo- and heterodimers, and even oligomers, and are expressed in tissue and cell-specific manner, suggesting that each S100 protein may perform different functions [3]. Indeed, it is well documented that S100 proteins are involved in several biological processes, such as cell cycle regulation, cell growth, cell differentiation and motility through a broad range of intracellular and extracellular activities [4-6]. Intracellular functions include regulation of protein calcium homeostasis, phosphorylation, regulation of cytoskeletal components and regulation of transcriptional factors. Extracellularly they act in a cytokine like manner through the receptor for advanced glycation end products (RAGE) [7].

The association between S100 family members and tumors may be explained by several observations: firstly, the region of human chromosome 1q21, where most of S100 genes are clustered, is prone to genomic rearrangements, likely supporting the tumor progression [8]; secondly, several S100 members show altered expression levels in cancer cells compared to normal cells and are differentially expressed in various malignancies, according to types and stages of cancer [9-15]. Finally, a number of S100 proteins have been shown to interact with and to regulate various proteins involved in cancer and exert different effects on p53 activity [16-20]. However, the occurrence, the role and the possible coordination of this group of proteins in breast cancer is still poorly known. In this study we describe a large-scale proteomic investigation performed on breast cancer patients for the screening of multiple forms of S100 proteins. The results have shown that the majority of S100 proteins were present at very low levels, if not absent, in the non-tumoral tissues adjacent to the primary tumor. The proteomic screening, extended to 100 cryo-preserved breast cancer tissues, showed that some S100 protein members were ubiquitously expressed in almost all patients, while others appeared more sporadic among the same group of patients. Most, if not all, of the detected S100 members appeared reciprocally correlated. More interestingly, patients which developed distant metastases after a three year follow-up showed a general tendency of higher S100 protein expression, compared to the disease-free group.

## Methods

### Clinical specimens

The present study was conducted on 100 surgical tissues of ductal infiltrating breast cancer collected between 2003 and 2007 in the Breast Unit of the La Maddalena Hospital. Research was carried out in compliance with the Helsinki Declaration with the patients' written consent and with the approval of the Institutional Review Board (N°515/2008) from the La Maddalena Hospital. The study used leftover specimens, that is, aliquots of specimens collected for routine clinical care, and immediately frozen at -80°C until used. The specimens were not individually identifiable.

The patients of this study did not receive any cytotoxic/endocrine treatment prior to surgery. Diagnosis of ductal breast cancer (G2/G3) was confirmed histopathologically.

Post**-**operative monitoring to define whether or not distant metastases were present, was performed by conventional imaging follow-up, consisting of chest radiography, bone scintigraphy, magnetic resonance imaging (MRI) and positron emission tomography using 2-[fluorine-18]fluoro-2-deoxy-D-glucose (FDG-PET), as clinically indicated at La Maddalena Hospital.

### Sample preparations

The frozen breast tissue samples were washed several times with phosphate-buffered saline and homogenized in RIPA buffer (50 mM Tris pH 7.5, 0.1% Nonidet P-40, 0.1% deoxycholate, 150 mM NaCl, 4 mM EDTA) containing a mixture of protease inhibitors (0.01% aprotinin, 10 mM sodium pyrophosphate, 2 mM sodium orthovanadate, 1 mM PMSF). The extraction was carried out overnight at 4°C with the same buffer. The total cellular lysate was centrifuged to remove tissue debris, and the resulting supernatant dialysed against ultrapure distilled water, lyophilized and stored at -80°C until use. The total protein concentration was determined by the Bradford method using bovine serum albumin as a standard [21].

### Two Dimensional Gel Electrophoresis

The proteins extracted from breast cancer tissue and normal adjacent tissue were solubilised in a buffer containing 4% CHAPS, 40 mM Tris, 65 mM DTE in 8 M urea. Aliquots of 45 μg (analytical gels) or 1.5 mg (preparative gels) of total proteins were separately mixed with 350 μL of rehydration solution containing 8 M urea, 2% CHAPS, 10 mM DTE and 0.5% carrier anpholytes (Resolyte 3.5-10), and applied for IEF using commercial sigmoidal IPG strips, 18 cm long with pH range 3.0-10. The second dimension was carried out on 9-16% linear gradient polyacrylamide gels (SDS-PAGE), and the separated proteins were visualized by ammoniacal silver staining. Stained gels were digitized using a computing densitometer and analyzed with Image Master software (Amersham Biosciences, Sweden). Gel calibration was carried out using an internal standard and the support of the ExPaSy molecular biology server, as described [22].

### Protein identification

N-Terminal microsequencing was performed by automated Edman degradation in a protein sequencer (Procise, 419 Applied Biosystems), as previously described [23].

Mass spectrometric sequencing was performed by Voyager DE-PRO (Applied Biosystems) mass spectrometer as described [24]. Briefly, proteins were digested using sequencing-grade trypsin (20 *μ*g/vial). The tryptic peptide extracts were dried and redissolved in 10 *μ*L of 0.1% trifluoroacetic acid (TFA). The matrix, R-cyano-4-hydroxycinnamic acid (HCCA), was purchased from Sigma-Aldrich. A saturated solution of HCCA (1 *μ*L) at 2 mg/200 *μ*L in CH3CN/H2O (50:50 (v/v)) containing 0.1% TFA was mixed with 1 *μ*L of peptide solution on the MALDI plate and left to dry. MALDI-TOF mass spectra were recorded in the 500-5000 Da mass range, using a minimum of 100 shots of laser per spectrum. Delayed extraction source and reflector equipment allowed sufficient resolution to consider MH+ of monoisotopic peptide masses. Internal calibration was done using trypsin autolysis fragments at *m*/*z *842.5100, 1045.5642, and 2211.1046 Da. Peptide mass fingerprinting was compared to the theoretical masses from the Swiss-Prot or NCBI sequence databases using Mascot http://www.matrixscience.com/. Typical search parameters were as follows: (50 ppm of mass tolerance, carbamidomethylation of cysteine residues, one missed enzymatic cleavage for trypsin, a minimum of four peptide mass hits was required for a match, methionine residues could be considered in oxidized form.

### Western Blotting

For immune detection the 1D-gels were electrotransferred onto nitrocellulose membrane (HyBond ECL, Amersham) and stained with Ponceau S (Sigma). The membranes were then probed with one of the following monoclonal antibodies: anti-actin (Oncogene), anti-S100A2, anti-S100A4, anti-S100A6, anti-S100A7, anti S100A8 (Santa Cruz), or polyclonal antibodies: anti-S100A11, anti-S100A13 (SantaCruz). Following incubation with the appropriate peroxidase-linked antibody, the reaction was revealed by the ECL detection system, using high performance films (Hyperfilm ECL, Amersham).

### Quantification and normalization methods

Quantitative expression levels were calculated as the volume of the spots (i.e. integration of optical density over the spot area). In order to correct for differences in gel staining, spot volumes relative to the sum of the volume of all spots an each gel (%Vol) were calculated by the software.

**Figure 1:**
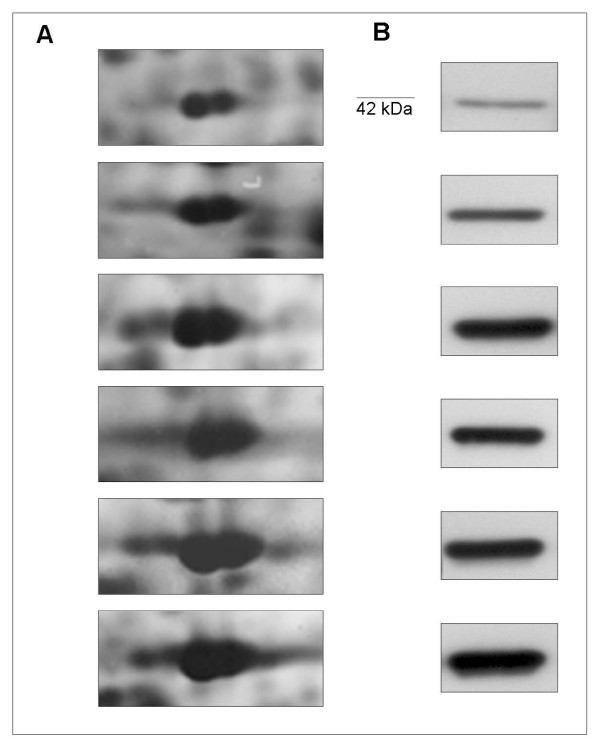
**Representative group of tissue extracts proteomics assayed for the actin content**. A) Experimental windows cropped from Image-Master 2 D Platinum software containing the sections of actin spots from the silver stained 2D-IPG. B) 1D-western blot validation of the actin content on corresponding tissue extracts with the same gel-loading.

Since the cell densities within an area of the surgical sample, may be very variable among the different patients, measurements of relative expression levels of individual protein spots were normalized in each map for actin content [24] and the final value was designated as N%V. The relative abundance of silver stained actin was validated by western blot assays on the same tissue extract (Fig. 1).

For statistical analyses Ms Excel and Graph Pad Prism 4 software were used. Correlation of S100 protein members for breast cancer patients was performed using the Pearson correlation test. The difference in S100 expression between metastatic versus disease free patients was analyzed by unpaired F test. In all cases, p < 0.05 was considered significant (*), p < 0.01 highly significant (**) and p < 0.001 very highly significant (***).

## Results

### Proteomic identification of S100 proteins

**Figure 2:**
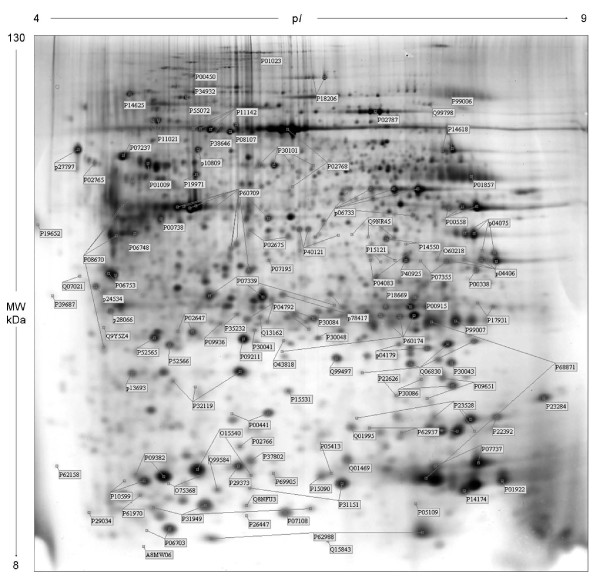
**Representative gel image of a breast cancer tissue**. 2-D separation was performed on IPG gel strips (18 cm, 3.0-10 NL) followed by the SDS-PAGE on a vertical linear-gradient slab gel (9-16%T). Protein spots of known identity are marked with the Swiss-prot accession number. When present, different isoforms of the same protein were jointly labelled.

In a previous work we reported the comparative proteomic profiles of proteins from 37 breast cancer surgical tissues [24]. Fig. 2 shows an updated proteomic map representative of a breast surgical tissue. The identified proteins are marked with labels corresponding to the access number of the Swiss-Prot database: 205 protein spots, corresponding to 114 distinct proteins, were identified in the maps. The protein identity was assessed by Maldi-Tof or N-Terminal microsequencing.

**Figure 3:**
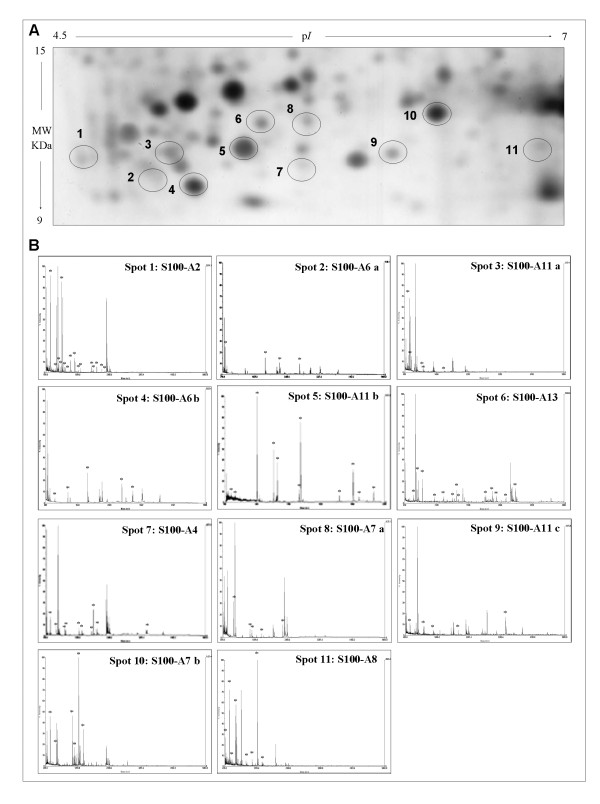
**Identification of S100 proteins**. A) Gel window comprising an area covering a pI/kDa range of 4.5-7/15-9 kDa, where the majority of known members of non-keratin associated S100 proteins, are expected to localize. The protein spots localizing in this area were picked from the gels and digested with trypsin. The resulting fragments were analyzed by mass spectrometry. Eleven spots were identified as S100 protein members. The numbers indicate the eleven spots corresponding to S100 proteins, whose spectra are shown in B). The mass peaks marked with an asterisk match the theoretical spectrum of the assigned proteins.

Fig. 3A shows the image of a gel window comprising an area covering a pI/kDa range of 4.5-7/15-9 kDa, where the majority of known members of S100 proteins are expected to localize.

After a comprehensive screening of protein spots included in this area, in different proteomic maps, we identified the following S100 protein members: S100A2 (protein S-100L), S100A4 (metastasin), S100A6 (Calcyclin, Prolactin receptor-associated protein), two isoforms, S100A7 (psoriasin), two isoforms, S100A8 (Calgranulin-A), S100A11 (Calgizzarin), three isoforms and S100A13 (S100 calcium-binding protein A13). Different isoforms of the same protein were labelled by alphabetic letters starting from the more acidic one. Fig. 3B illustrates the Maldi-Tof mass spectra of the corresponding spots shown in Fig. 3A. The list of identified proteins is shown in Table 1.

**Table 1:** Synopsis of the information on the identified S100 proteins.

SPOT NUMBER	ENTRY NAME	SPOT NAME	AC	PROTEIN NAME	MOWSE SCORE	NUMBER OF MASS VALUES SEARCHED	NUMBER OF MASS VALUES MATCHED	(%) SEQUENCE COVERAGE	THEORETICAL MW (Da) - pI
1	S10A2	S100A2	P29034	S100-A2	229	18	16	59	11337-4.68
7	S10A4	S100A4	P26447	S100-A4 Metastasin	129	16	10	51	11949-5.85
2	S10A6	S100A6 a	P06703	S100-A6 Calcyclin	77	4	4	28	10230-5.33
4	S10A6	S100A6 b	P06703	S100-A6 Calcyclin	68	5	4	40	10230-5.33
8	S10A7	S100A7 a	P31151	S100-A7 Psoriasin	80	8	5	39	11470-6.27
10	S10A7	S100A7 b	P31151	S100-A7 Psoriasin	101	6	6	35	11470-6.27
11	S10A8	S100A8	P05109	S100-A8 Calgranulin	76	36	8	49	10885-6.51
3	S10AB	S100A11 a	P31949	S100-A11 Calgizzarin	82	5	5	42	11847-6.56
5	S10AB	S100A11 b	P31949	S100-A11 Calgizzarin	127	11	11	61	11847-6.56
9	S10AB	S100A11 c	P31949	S100-A11 Calgizzarin	73	6	5	34	11847-6.56
6	S10AD	S100A13	Q99584	S100-A13	226	19	13	89	11464-5.91

Entry names, protein names, accession numbers and theoretical MW and pI are from the Swiss-Prot database. Mowse score represent -10* Log (P), where P is the probability that the observed match is a random event. Number of mass value matched and the % of sequence coverage are given by Mascot database in the protein view session.

### S100 proteins are preferentially expressed in the tumor mass

**Figure 4:**
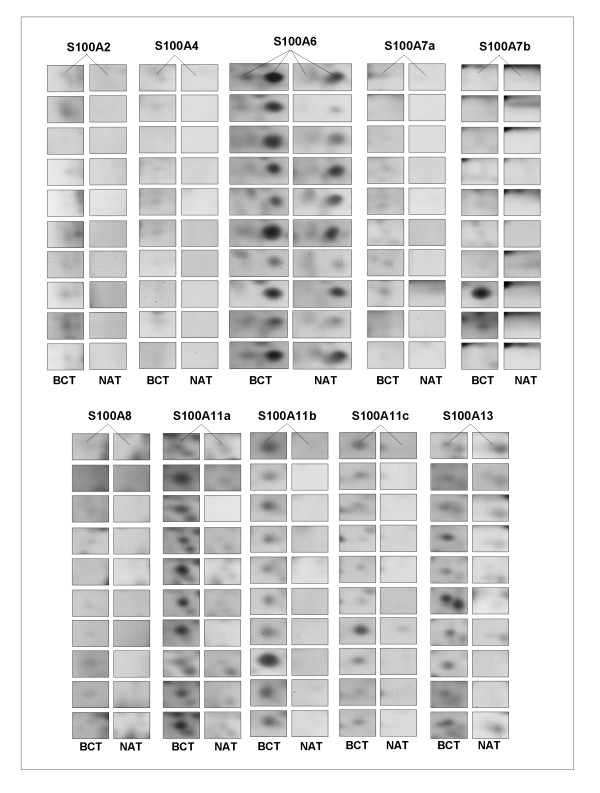
**Panel of cropped areas of individual S100 protein spots from matched breast cancer tissues (BCT) and non tumoral adjacent tissues (NAT)**. The experiments were conducted on a pilot group of 10 patients, selected for the present study.

**Figure 5:**
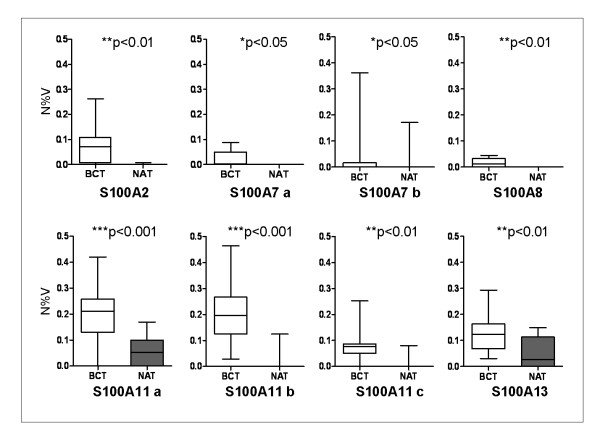
**Quantitative analysis of S100 proteins, given as box-plot graphs, of matched breast cancer tissues (BCT) and non tumoral adjacent tissues (NAT)**. Quantification was deduced by the 2 D gels, analyzed by Image-Master software. In ordinate are the values of N%V. Statistical significance was analyzed by the Student's t-test: *p < 0.05 was considered significant; **p < 0.01 highly significant; ***p < 0.001 very highly significant. The data in the graphs are expressed as median ±SD.

A group of 10 breast cancer tissues and their matched non tumoral counterparts were analyzed for comparative proteomic expression of the S100 proteins. Fig. 4 shows a panel of the cropped images from 2-D matched gels of the 10 selected patients. The S100 proteins are almost exclusively present in the tumor extracts, even though some S100 members are expressed at low, or very low levels, for example S100A2, S100A4 and S100A8. Fig 5 shows box-plot graphs illustrating the quantitative variation of S100 protein expression levels between breast cancer and normal adjacent tissues. Significant differences were observed for all S100 protein members, except for S100A4 (expressed only in 5 patients) and for S100A6 (expressed also in normal tissues). Therefore these two proteins were not included in Fig. 5.

### Proteomic distribution of S100 proteins within a cohort of 100 breast cancer patients

**Figure 6:**
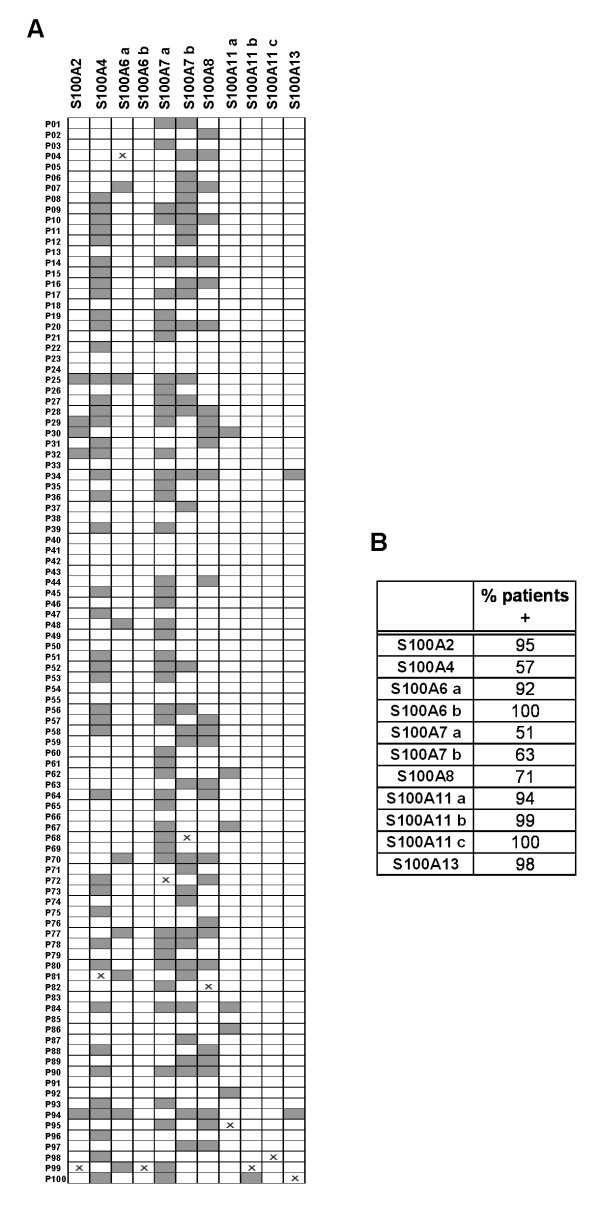
**Qualitative analysis of the eleven S100 protein forms among the 100 patients**. (A) Diagram illustrating the occurrence of the identified S100 protein members (abscissa) among the patients indicated from P01 to P100 (ordinate). The gray boxes indicate absence of proteins in the map of the corresponding patient; the crosses in the white boxes indicate the patients expressing the given protein at its highest level among the others. (B) Table reporting the percentage of the enrolled patients expressing the different S100 protein forms.

Fig. 6A shows a diagram illustrating the occurrence of the identified S100 protein members (abscissa) among the 100 patients indicated from P01 to P100 (ordinate). The gray boxes indicate the absence of proteins in the map of the corresponding patient and the crosses indicate the patient expressing the given protein at its highest level. Interestingly, some proteins are expressed in almost all patients (S100A6, S100A11, S100A2 and S100A13); while others are expressed in a variable number of patients, i.e.: S100A8 in 71% of the patients, S100A4 in 57% and S100A7 in 51% (isoform *a*) and 63% (isoform *b*) (Fig. 6B).

**Figure 7:**
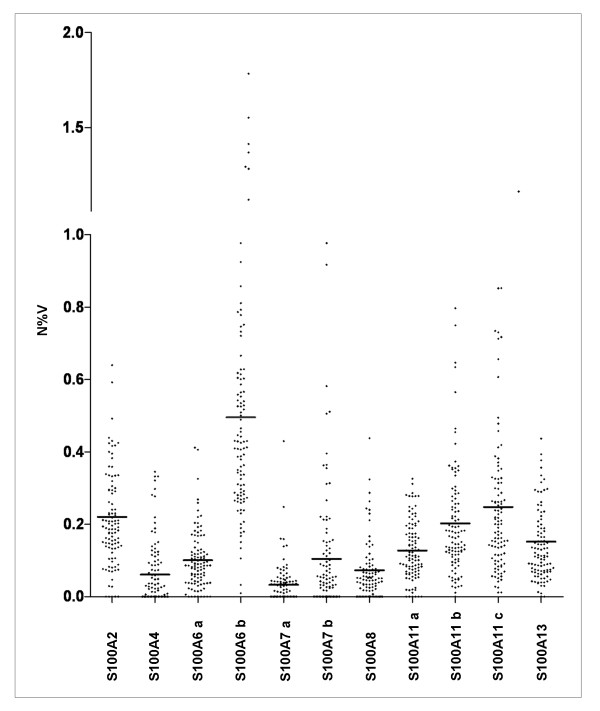
**Diagram of the relative intensities of the eleven S100 protein forms among the 100 patients**. In abscissa are indicated the protein names and in the ordinate the density values. Bars indicate the mean of each protein expression level among patients.

In order to quantify the relative expression levels of individual S100 protein members the intensity of each protein spot was normalized for the actin content of the corresponding map. Fig. 7 shows the expression levels of each S100 protein, including isoforms, within the cohort of 100 patients. Except for S100A6, the average value of expression for each protein form (evaluated as N%V) does not exceed the relative abundance value of 0.25. The expression range of each S100 member is quite variable among patients: for instance, while the basic form of S100A6 ranges from 0.009 to 1.7, S100A4 ranges from 0.005 to 0.35.

### Relationship between expression levels of S100 members

**Figure 8:**
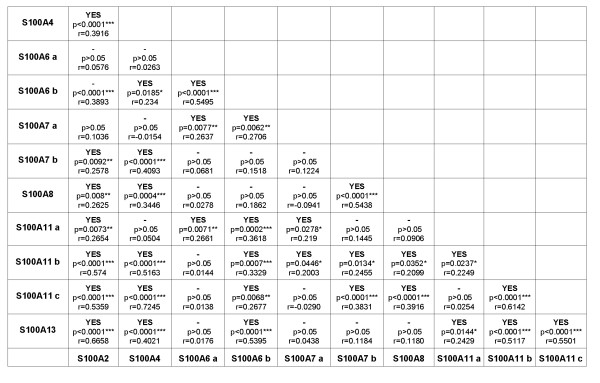
**Association analysis of expression levels of S100 members evaluated as N%V**. Statistical significance of the S100 members correlation was assessed by Pearson test and considered as significant, highly significant and very highly significant (*p < 0.05; **p < 0.01; ***p < 0.001). Correlation is indicated as Yes.

The expression level of each S100 protein was cross-tabulated with the other protein members and statistical significance was assessed by the Pearson test. A significant association was observed for a high percentage of them (Fig. 8). More analytically, for S100 protein spots present as multiple isoforms, the more basic ones, having the closest p*I *to the theoretical values and likely representing the primary gene product, showed positive correlations with the other S100 members, except for S100A7 b and S100A8 that showed no correlation with the b-isoform of the S100A6 and with the S100A13.

### Western blot validation of S100 proteins

**Figure 9:**
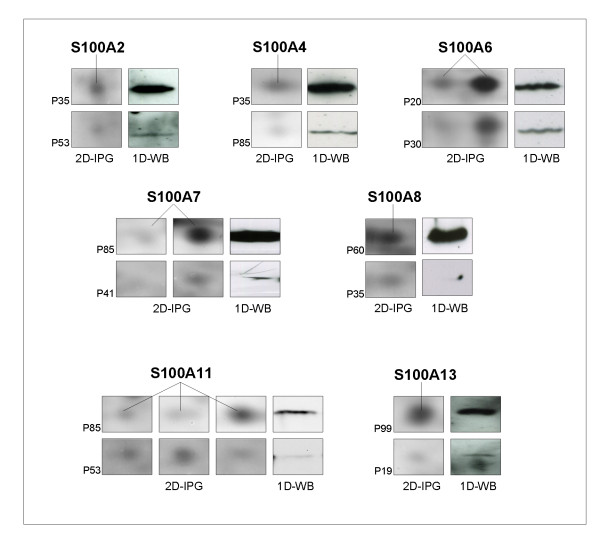
**Western Blot validation of S100 proteins detected by silver stain proteomics**. The panel shows the cropped areas of 2 D gels containing the silver stained S100 protein spots (left), paired with the 1D-western blot image on the same selected breast cancer tissue (right). The validation was performed on two surgical samples for each S100-antibody, chosen among the 100 patient tissues showing respectively high and low levels of each S100 protein.

Immunological assays were performed to confirm the differential expression of all the S100 proteins identified in 2D-IPG. Validation with the appropriate antibodies, was performed on patient couples, chosen among the ones indicated by P01-P100 in the diagram in Fig. 5A, having high and low levels of the S100 proteins, respectively. Fig. 9 shows a panel of cropped 2 D gels containing the silver stained S100 protein spots, paired with the 1D-western blot image on the same tissue extract.

### Association of S100 members with tumor variables

The expression levels of S100 proteins were correlated with current clinical-pathological parameters which included age, tumor size, nodal status, immuno-cytochemical presence of HER-2, oestrogen receptors, progesterone receptor, and Ki67 (Table 2). The results showed no significant correlations of the different S100 protein forms with tumor variables, except for S100A6 (isoform b) and S100A13 correlating with Ki67 (p = 0.043; p = 0.041) and for S100A11 (isoform a) correlating with nodal status (p = 0.021).

**Table 2:** Clinical-pathological characteristics of patients and correlation with S100 protein expression.

		S100A2	S100A4	S100A6 a	S100A6 b	S100A7 a	S100A7 b	S100A8	S100A11 a	S100A11 b	S100A11 c	S100A13
**Age (n = 100)**		p > 0.05	p > 0.05	p > 0.05	p > 0.05	p > 0.05	p > 0.05	p > 0.05	p > 0.05	p > 0.05	p > 0.05	p > 0.05
*≤ 56*	42%											
*> 56*	58%											
**T (n = 73)**		p > 0.05	p > 0.05	p > 0.05	p > 0.05	p > 0.05	p > 0.05	p > 0.05	p > 0.05	p > 0.05	p > 0.05	p > 0.05
*1*	33%											
*2*	56%											
*3*	5%											
*4*	6%											
**N (n = 76)**		p > 0.05	p > 0.05	p > 0.05	p > 0.05	p > 0.05	p > 0.05	p > 0.05	p = 0.0210*	p > 0.05	p > 0.05	p > 0.05
*Neg*	39%											
*Pos*	61%											
**HER-2 (n = 80)**		p > 0.05	p > 0.05	p > 0.05	p > 0.05	p > 0.05	p > 0.05	p > 0.05	p > 0.05	p > 0.05	p > 0.05	p > 0.05
*0*	45%											
*1*	24%											
*2*	14%											
*3*	17%											
**ER (n = 90)**		p > 0.05	p > 0.05	p > 0.05	p > 0.05	p > 0.05	p > 0.05	p > 0.05	p > 0.05	p > 0.05	p > 0.05	p > 0.05
*Neg*	31%											
*Pos*	69%											
**PR (n = 89)**		p > 0.05	p > 0.05	p > 0.05	p > 0.05	p > 0.05	p > 0.05	p > 0.05	p > 0.05	p > 0.05	p > 0.05	p > 0.05
*Neg*	42%											
*Pos*	58%											
**Ki67% (n = 77)**		p > 0.05	p > 0.05	p > 0.05	p = 0.043*	p > 0.05	p > 0.05	p > 0.05	p > 0.05	p > 0.05	p > 0.05	p = 0.041*
*≤ 15%*	23%											
*16-30%*	29%											
* > 30%*	48%											

Pearson t-test was used to evaluate the association among age, T, N, HER-2, ER, PR and Ki67 and S100 proteins expression. *p < 0.05 was considered significant and marked in bold.

### Association of S100 members with metastases

**Figure 10:**
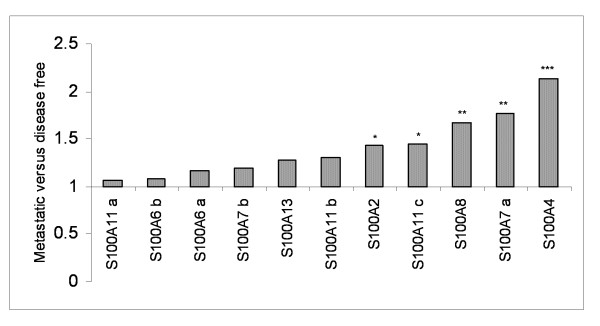
**Changes in S100 expression levels between metastases and disease free -related patients**. Data are represented as ratio of metastatic group to disease free group. P-values, calculated by F-test were considered as significant, highly significant and very highly significant (*p < 0.05; **p < 0.01; ***p < 0.001).

From several reports, individual S100 proteins have been found to correlate with metastasis; however a wide-ranging pattern of S100 protein members in a large scale of breast cancer patients was never screened before. Therefore, we analyzed our data set concerning the expression level of S100 proteins with respect to their association with the development of distant metastases. Patients with 3-year follow-up were fifty seven, 22 had developed distant metastases while 35 were disease-free. As shown in Fig. 10 the expression level of each S100 protein (expressed as average among patients of each group) was 1.07 to 2.13 fold higher in the metastatic group. The unpaired F-test statistical method was used to detect expression variance among the S100 proteins in metastatic patients compared with the disease free group.

## Discussion

The clinical interest for S100 proteins as putative cancer biomarkers is continuously expanding. Major reasons for this are their multifunctional properties with a regulatory role in a variety of cellular and extracellular processes, and also the clustering of the majority of the S100 genes on a region of chromosome 1q21 which are often rearranged in cancer.

Although there are numerous reports on the correlation or involvement of individual S100 members in cancer [25-29], to our knowledge this study is the first to screen contextually for several members of the protein family through a large-scale proteomic approach. Proteomics is presently the only system able to detect protein isoforms of potential interest, which are not detectable by gene expression or immunohistochemical investigations [30-34]. Collectively we have identified eleven S100 protein forms, corresponding to 7 protein members. The first goal of this study was the finding of the almost exclusive expression of S100 members in cancerous breast tissues compared with normal adjacent tissues, an observation that *per se *substantiates the role of S100 proteins as putative biomarkers. This observation is in good agreement with literature data supporting the evidence that altered expression of many of S100 members occurs in several cancers including breast, lung, kidney, bladder, gastric, thyroid, prostate and oral cancers [see for review [29]].

The second goal of our study was the quali-quantitative proteomic screening of a significant number of the S100 family proteins among a large group of 100 breast cancer patients, all diagnosed as ductal infiltrating carcinomas. Qualitative analysis showed that some of the S100 protein members are ubiquitously expressed in all patients while others appeared more sporadic. Among the first, are: S100A2, S100A6, S100A11 and S100A13 (all isoforms, when present); the members with more or less sporadic appearance are: S100A8 (71%), S100A4 (57%) and S100A7 (51%, isoform *a *and 63%, isoform *b*).

The quantitative evaluation showed that the expression levels of each S100 member was different among patients, but collectively, most of the S100 protein forms were statistically correlated. This adds complexity to the role of this protein category in breast cancer and suggests a possible common pathway of (dys-)regulation.

Finally, we investigated the prognostic potential of S100 proteins to predict distant metastatic relapse during a time lapse of three years from the surgical intervention. The most robust correlation with metastasis regarded primarily the protein S100A4, and secondly the protein S100A7. The S100A4, also named metastasin for its presumed role in metastasis promotion, is one of the most investigated in the recent literature [see for review [35]], as a promising biomarker of breast metastasis. Our results are in agreement with this hypothesis, since S100A4 expression level shows an increase of more than 2 fold in the metastatic group.

## Conclusions

Present data strongly support the hypothesis that a significant deregulation of multiple S100 family members is associated with breast cancer progression, and suggest that these proteins might act as potential prognostic factors for patient stratification. Although the patho-physiologic implications of the S100 proteins in cancer still require further clarification, the description of their differential occurrence in a large group of breast cancer patients, at proteomic levels, is a further important step promoting advancement of scientific knowledge for biomarker application in clinical practice.

## Competing interests

The authors declare that thet have no competing interests.

## Authors' contributions

All authors participated in the interpretation and elaboration of the findings.

IPM and PC were responsible for the conception and design of the study and drafted the manuscript. GDC performed the protein identification. NNA performed protein analysis. FC, MRM, RM carried out 2D-IPG. CL and ER contributed with clinical information. All authors have read and approved the final manuscript.

## Pre-publication history

The pre-publication history for this paper can be accessed here:

http://www.biomedcentral.com/1471-2407/10/476/prepub
